# Acute pulmonary thromboembolism caused by factor V Leiden mutation in South Korea: A case report

**DOI:** 10.1097/MD.0000000000016318

**Published:** 2019-07-12

**Authors:** Hun Jee Choe, Koung Jin Suh, Ji Yun Lee, Minyoung Kim, Man Jin Kim, Sung Sup Park, Ji-Won Kim, Se Hyun Kim, Jin Won Kim, Jeong-Ok Lee, Yu Jung Kim, Keun-Wook Lee, Jee Hyun Kim, Soo-Mee Bang, Jong Seok Lee

**Affiliations:** a Department of Internal Medicine, Seoul National University, Seoul National University Bundang Hospital, Seongnam; b Department of Laboratory Medicine, Seoul National University Hospital, Seoul National University College of Medicine, Seoul, Republic of Korea.

**Keywords:** factor V Leiden, thrombophilia, venous thromboembolism

## Abstract

**Rationale:**

Although Factor V Leiden (FVL) mutation is a major cause of inherited thrombophilia in Western populations; the mutation is extremely rare in Asia.

**Patient concerns:**

Here we report a case of a 28-year old Korean woman admitted to our hospital with extensive pulmonary embolism.

**Diagnosis:**

She was heterozygous for FVL mutation up on evaluation, and screening for asymptomatic family members also revealed heterozygous FVL mutation for her mother.

**Interventions:**

Enoxaparin 1 mg/kg was initiated, followed by rivaroxaban 15 mg every 12 hours.

**Outcomes:**

The patient showed improvement in both subjective dyspnea and right ventricular dysfunction and was successfully discharged after five hospital days.

**Lessons:**

FVL mutation screening may be considered in Asian patients with thrombophilia of uncertain etiology in the future.

## 1 Introduction

Venous thromboembolism (VTE), which constitutes pulmonary embolism (PE) and deep vein thrombosis (DVT), is an important public health concern with high morbidity and mortality requiring hospitalization.^[1]^ It is therefore essential to undergo a comprehensive evaluation for the cause of VTE in patients with first occurrence. Major risk factors for VTE include surgery, active cancer, immobility, trauma or fracture, pregnancy, and estrogen therapy.^[2]^ Hereditary deficiencies such as antithrombin (AT), protein C (PC), or protein S (PS), as well as Factor V Leiden (FVL) or prothrombin G20210A mutations have been well-established risk factors for thrombophilia of genetic origin.^[3,4]^ FVL thrombophilia is the most prevalent genetic mechanism for inherited hypercoagulable states among the general Caucasian population, with 3% to 7% of the population harboring the mutation.^[5]^ However, FVL thrombophilia has previously not been reported in East Asians.^[6]^ We herein report the first case of inherited heterozygous FVL mutation in South Korea.

## 2 Case report

A 28-year old Korean woman presented to the emergency department after a witnessed syncopal episode on July 2017. She had epigastric discomfort and experienced dyspnea on exertion upon climbing stairs 2 days before admission. On the day of admission, she had transiently lost consciousness while complaining of dizziness. Her previous medical history was unremarkable. History of smoking tobacco, alcohol, or drug abuse was denied. Upon further inquiry, she admitted to having taken oral contraceptive pills for 5 days before going on a trip.

**Figure 1:**
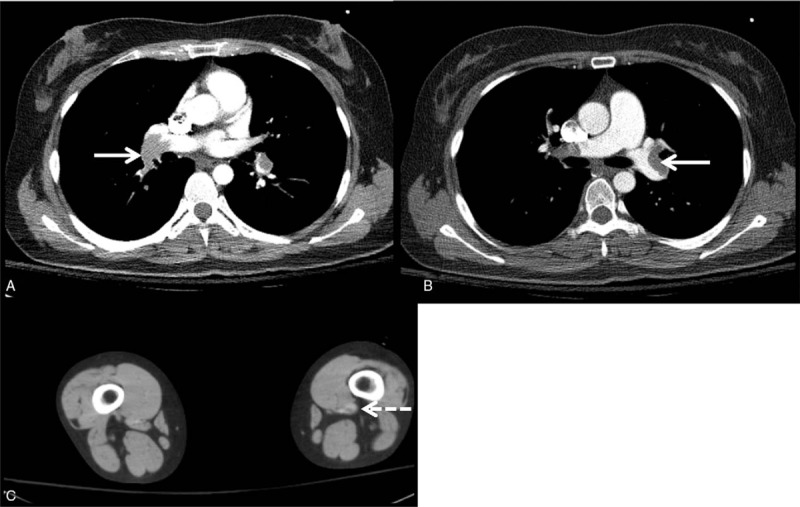
Contrast-enhanced computed tomography at initial visit. (A, B) Thromboembolism in both main pulmonary arteries and upper, middle, and lower lobar pulmonary arteries (*arrow* indicates the right and left pulmonary arteries, respectively) (C) Deep vein thrombosis in the left popliteal vein (*dotted arrow*).

On admission, she was alert and oriented but lethargic with initial blood pressure of 78/38 mm Hg, a pulse rate of 116/minutes, and oxygen saturation 76% while breathing ambient air. Cardiac examination showed regular tachycardia with accentuated S2 sound and wheezing, crackles were present in the lower lung field. Abdominal findings were unremarkable. There was no leg edema. Her electrocardiogram revealed sinus tachycardia, normal axis, and normal intervals. Arterial blood gas analysis results were as follows: pH 7.46, pCO2 31.2 mm Hg, pO_2_ 39.4 mm Hg, and bicarbonate 21.9 mmol/L. D-dimer was elevated to 10.1 μg/ml (reference range < 0.5 μg/ml). The complete blood count, electrolyte, glucose, prothrombin time, activated partial thromboplastin time, renal-, and liver-function tests were within normal range. A contrast-enhanced computed tomography (CT) scan was performed. There was near total occlusion of both main pulmonary arteries and upper, middle, and lower lobar pulmonary arteries that were consistent with acute pulmonary thromboembolism and deep vein thrombosis was seen at the left popliteal vein (Fig. 1). Echocardiography showed dilated right ventricle with dysfunction, D-shaped left ventricle and inferior vena cava dilatation without plethora.

The patient was transferred to the intensive care unit (ICU) for close monitoring. She was hemodynamically stabilized after aggressive fluid resuscitation without need for thrombolysis or embolectomy, and supplemental oxygen was discontinued after several days. Anticoagulation treatment with low-molecular weight heparin was initiated and she was successfully discharged on hospital day 5 after switching to a direct oral anticoagulant (DOAC), rivaroxaban 15 mg every 12 hours.

**Figure 2:**
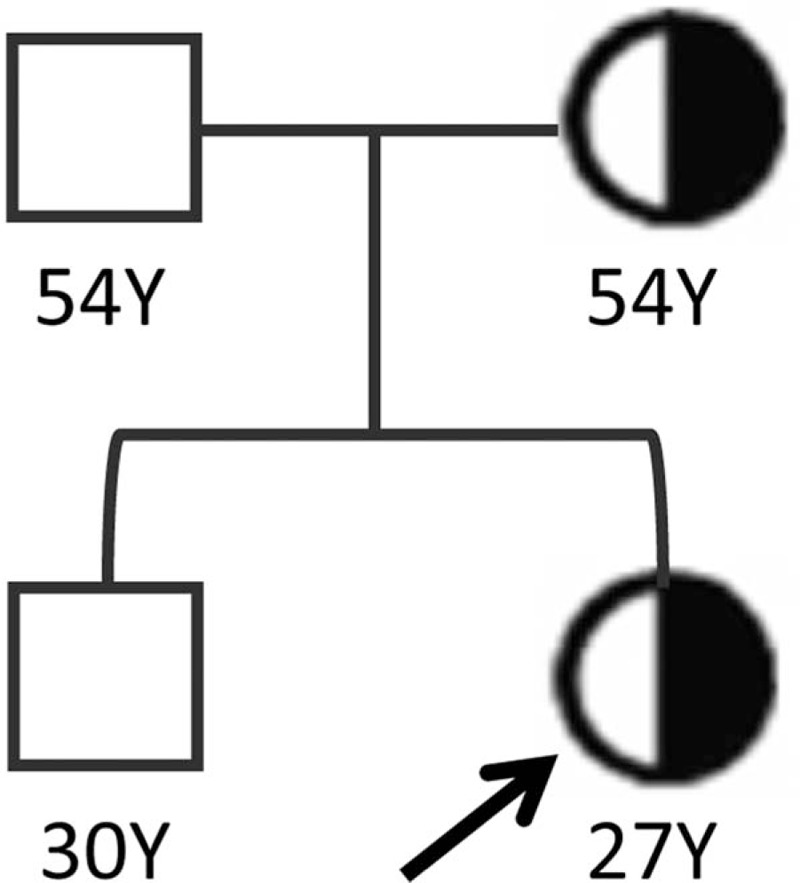
Pedigree analysis carrying the Factor V Leiden mutation. Men and women are shown as squares and circles, respectively. Half solid symbols represent individuals heterozygous for Factor V Leiden mutation. Our index patient is indicated with an arrow.

Thrombophilia study for the patient showed the following results: PC 103 IU/dl (reference range 70–130 IU/dl), PS 75 IU/dl (reference range 70–130 IU/dl), and AT III 95% (reference range 80–120%), all levels within normal range. Lupus anticoagulant, anticardiolipin antibodies, and prothrombin G20210A gene mutation were negative. Homocysteine level was 6.59 μmol/L (reference range 4–15 μmol/L) and factor VIII level 164% (reference range 52–192%) were within normal range. Multiplex PCR was carried out using SNaPshot system to screen for FVL. Screening for FVL showed heterozygous mutation (1691G > A), confirming the diagnosis for massive VTE due to FVL mutation. The patient's family was counselled, and further investigations were done for the patient's father, mother, and brother. The patient's mother was found to have FVL mutation, but other family members were found to be normal (Fig. 2).

Rivaroxaban was tapered down to a dose of 20 mg once daily after an initial 21-day course of higher dose therapy. Follow-up chest CT at 6 months of anticoagulation therapy showed no evidence of remnant pulmonary thromboembolism. After 12 months of anticoagulation therapy, rivaroxaban was discontinued. The patient is under close surveillance and has not had a subsequent thromboembolic event after discontinuation of rivaroxaban.

## 3 Discussion

FVL mutation is a major inheritable risk factor for thrombophilia in the Western population accounting for 20% to 25% of patients with VTE.^[7,8]^ However, the incidence of FVL has not been previously reported in Korea, Japan, or China until now.^[9,10]^ For it had never been reported in Korea before, the Korean Society of Thrombosis and Hemostasis does not recommend routine screening of FVL for patients suspected of inherited thrombophilia. To our knowledge, this is the first report of FVL thrombophilia in South Korea.

The risk of first time VTE is 3- to 7- fold higher in heterozygous carriers of FVL mutation and 50- to 100- fold in homozygous carriers compared to individuals without mutations.^[8,11,12]^ The clinical expression of FVL is influenced by coexisting risk factors. FVL interacts with external risk factors such as pregnancy, estrogen therapy, selective estrogen receptor modulators (SERMs), oral contraceptives, surgery, and travel.^[13]^ In this case, the concomitant use of oral contraceptives was considered to be a provoking factor for VTE, as it is known that the risk of VTE could increase by 30- to 60-fold in heterozygous FVL carriers taking oral contraceptives.^[14]^ Although PC, PS, and AT mutation tests on coexisting thrombophilic disorders were not conducted, their levels were within the normal range. Also, prothrombin G20210A gene mutation was negative.

Testing for thrombophilia in asymptomatic family members of patient with hereditary thrombophilia is not generally recommended.^[15]^ However, testing may be beneficial in female family members of patients with hereditary thrombophilia if results will influence choices regarding estrogen use or prophylaxis in the context of pregnancy.^[15]^ In this case, the mother of our patient turned out to be an asymptomatic heterozygous carrier, and now she may avoid going over estrogen therapy or using SERMs, which could increase the relative risk of thrombosis. Therefore, decisions regarding genetic screening test should be made on an individual basis.

Anticoagulation is the mainstay treatment of VTE. Many patients with inherited thrombophilia received anticoagulant therapy either over vitamin K antagonist (VKA) or a heparin product historically.^[16]^ The recently published 2016 American College of Chest Physicians (ACCP) guidelines suggest the use of DOACs over VKA for the treatment of VTE in patients without cancer.^[17]^ However, the role of DOACs in the treatment of inherited thrombophilia remains unknown.^[18]^ Wypasek et al reported 2 cases with VTE associated PS deficiency which showed resistance to anticoagulant effects of rivaroxaban.^[19]^ On the other hand, Cook et al showed that a patient with ovarian vein thrombosis associated FVL mutation has been successfully treated with rivaroxaban. Furthermore, a sub-group analysis by Schulman et al demonstrated that dabigatran was non-inferior to warfarin in patients with inherited thrombophilia in recurrent VTE or VTE-related deaths.^[20]^ In our case, there was no residual VTE in the repeated CT imaging 6 months after rivaroxaban treatment. Thus, DOACs may be suitable alternatives to VKA for patients with thrombophilia

There is limited information on genetic analysis of the proband's maternal ancestry that showed homogeneous ethnicity without involvement of Caucasian gene. Additional cases are required to elucidate whether FVL mutation could be regarded as one of the rare but plausible causes of genetic thrombophilia even in Eastern Asian populations.

## Acknowledgments

The authors thank the departments of Laboratory Medicine of the Seoul National University Hospital for providing help in the diagnosis and management of the patient described in the case report.

## Author contributions

**Conceptualization:** Hun Jee Choe, Ji Yun Lee.

**Data curation:** Hun Jee Choe, Ji Yun Lee, Minyoung Kim, Man Jin Kim, Sung Sup Park.

**Investigation:** Hun Jee Choe, Ji Yun Lee.

**Supervision:** Koung Jin Suh, Ji-Won Kim, Se Hyun Kim, Jin Won Kim, Jeong-Ok Lee, Yu Jung Kim, Keun-Wook Lee, Jee Hyun Kim, Soo-Mee Bang, Jong Seok Lee.

**Writing – original draft:** Hun Jee Choe, Ji Yun Lee.

**Writing – review & editing:** Hun Jee Choe, Ji Yun Lee.

Ji Yun Lee orcid: 0000-0002-5835-7219.
